# Supplementary material for: Targeting the fatty acid binding proteins disrupts multiple myeloma cell cycle progression and MYC signaling
Source: eLife. 2023 Mar 7;12:e81184. doi: 10.7554/eLife.81184 (PMC9995119; doi:10.7554/eLife.81184)
Supplement: Source data 1. [file elife-81184-data1.zip › WB/Western Blots.pptx]

## Slide 1
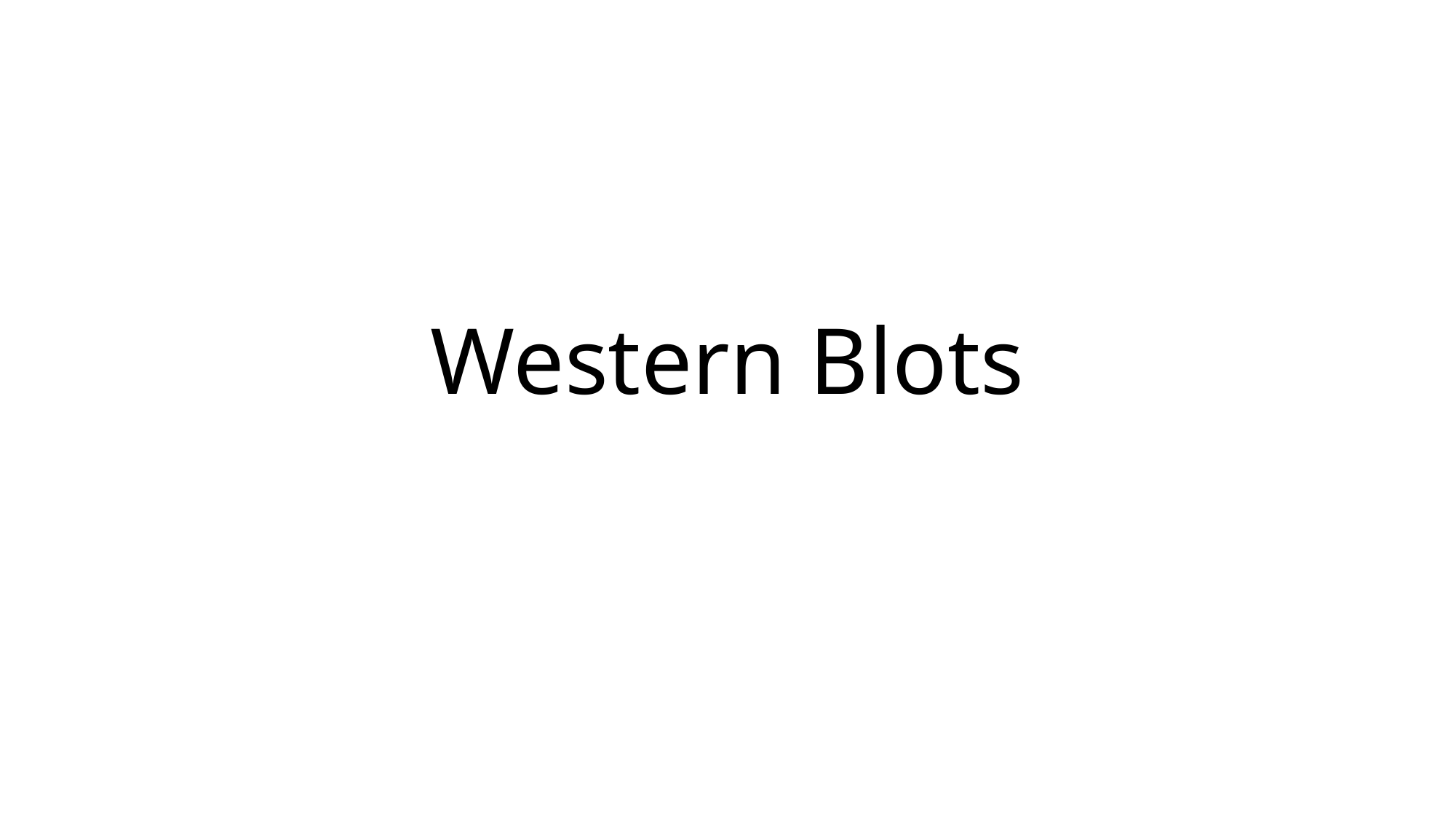

# Western Blots

## Slide 2
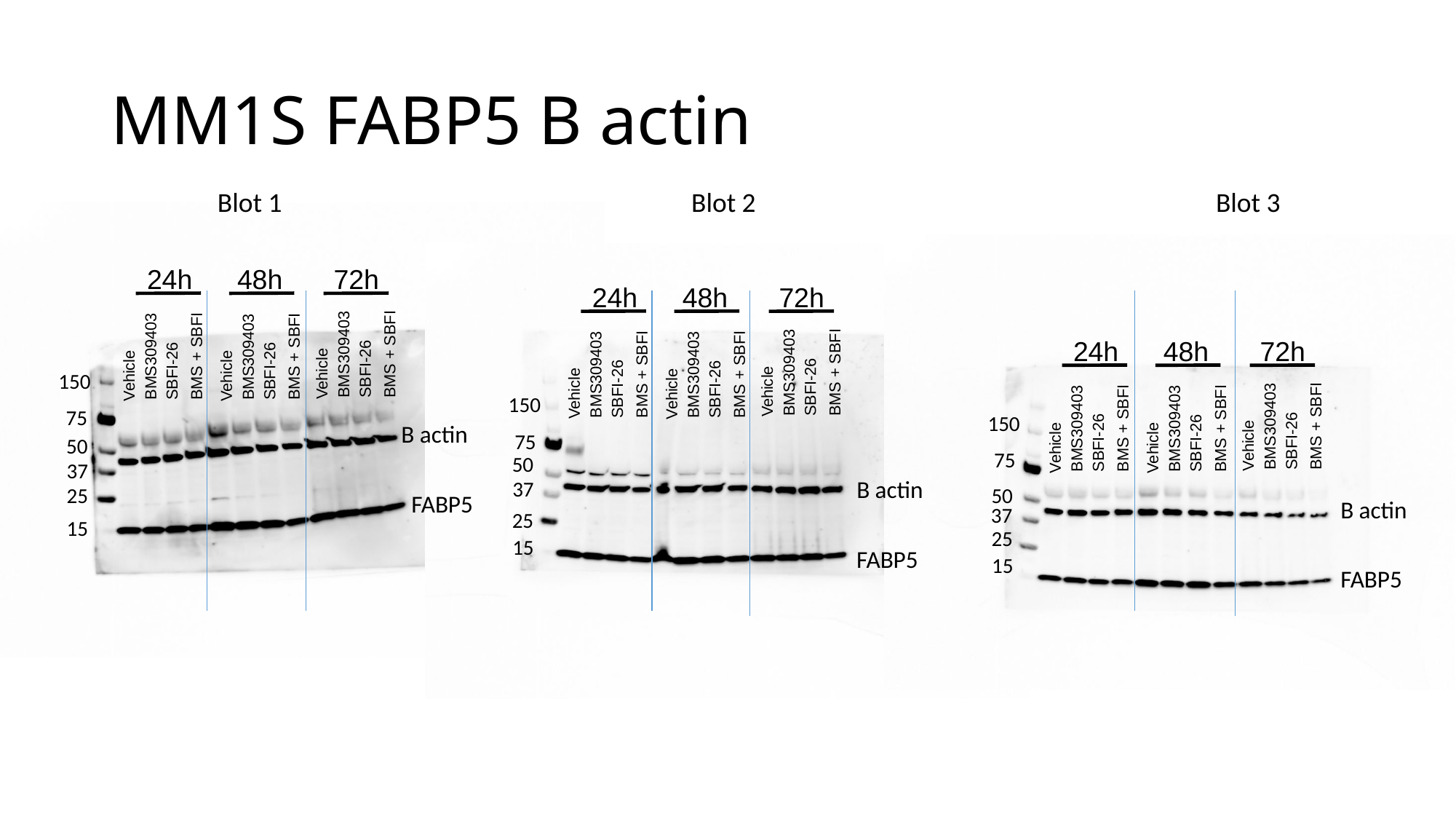

# MM1S FABP5 B actin
Blot 1
Blot 2
Blot 3
24h
48h
72h
24h
48h
72h
24h
48h
72h
BMS309403
BMS + SBFI
BMS309403
BMS + SBFI
BMS309403
BMS + SBFI
BMS309403
SBFI-26
BMS + SBFI
Vehicle
SBFI-26
SBFI-26
BMS309403
BMS + SBFI
Vehicle
BMS309403
BMS + SBFI
Vehicle
150
SBFI-26
Vehicle
SBFI-26
SBFI-26
Vehicle
Vehicle
150
75
BMS309403
150
BMS + SBFI
BMS309403
BMS + SBFI
BMS309403
BMS + SBFI
B actin
75
SBFI-26
Vehicle
SBFI-26
SBFI-26
Vehicle
Vehicle
50
75
50
37
B actin
37
25
50
FABP5
B actin
37
25
15
25
15
FABP5
15
FABP5

## Slide 3
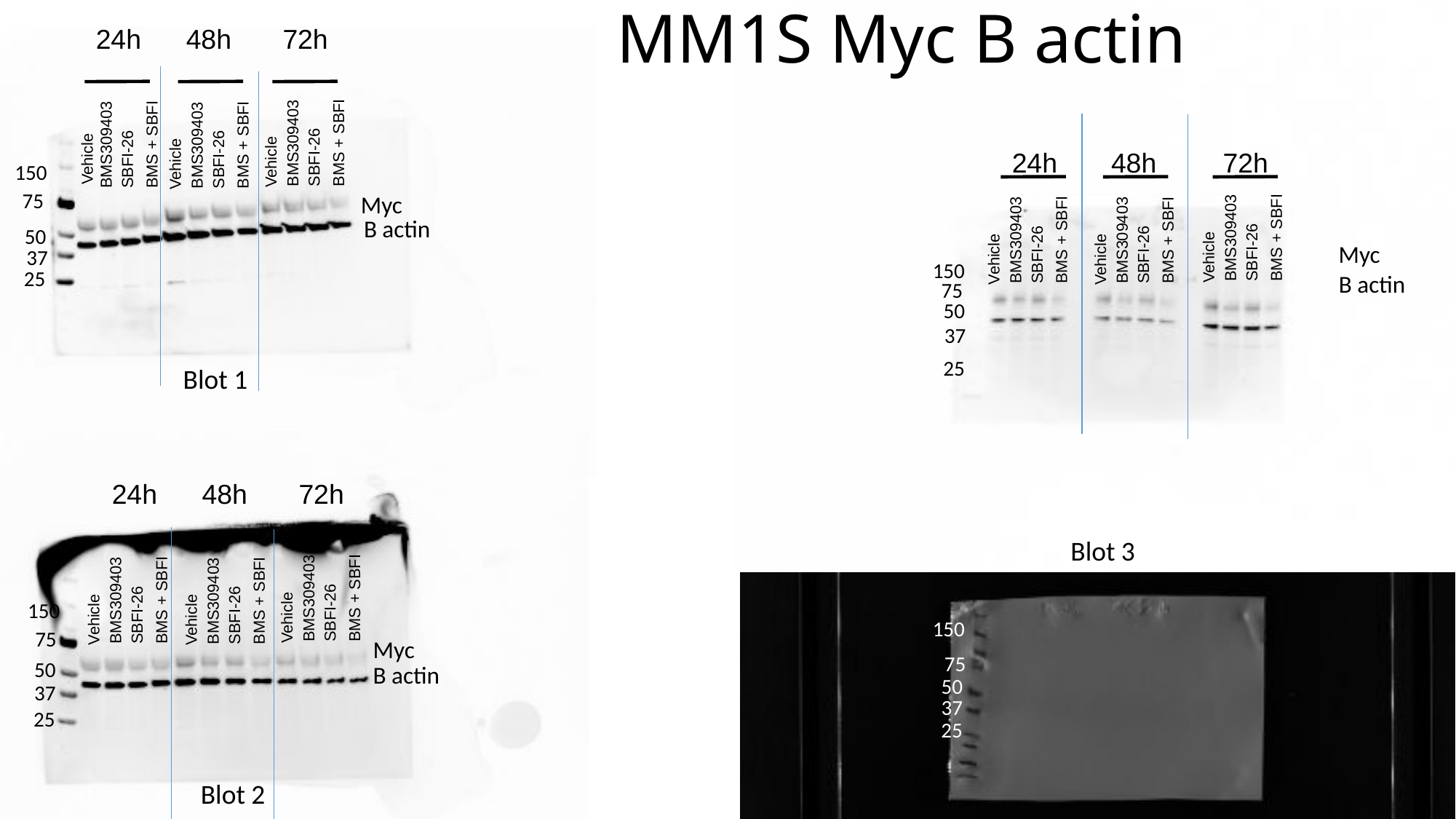

# MM1S Myc B actin
24h
48h
72h
BMS309403
BMS + SBFI
BMS309403
BMS + SBFI
BMS309403
BMS + SBFI
Vehicle
24h
48h
72h
SBFI-26
Vehicle
SBFI-26
SBFI-26
Vehicle
150
75
Myc
B actin
BMS309403
50
BMS + SBFI
BMS309403
BMS + SBFI
BMS309403
BMS + SBFI
Myc
SBFI-26
Vehicle
SBFI-26
SBFI-26
Vehicle
Vehicle
37
150
25
B actin
75
50
37
25
Blot 1
24h
48h
72h
Blot 3
BMS309403
BMS + SBFI
BMS309403
BMS + SBFI
BMS309403
BMS + SBFI
150
SBFI-26
Vehicle
SBFI-26
SBFI-26
Vehicle
Vehicle
150
75
Myc
75
50
B actin
50
37
37
25
25
Blot 2

## Slide 4
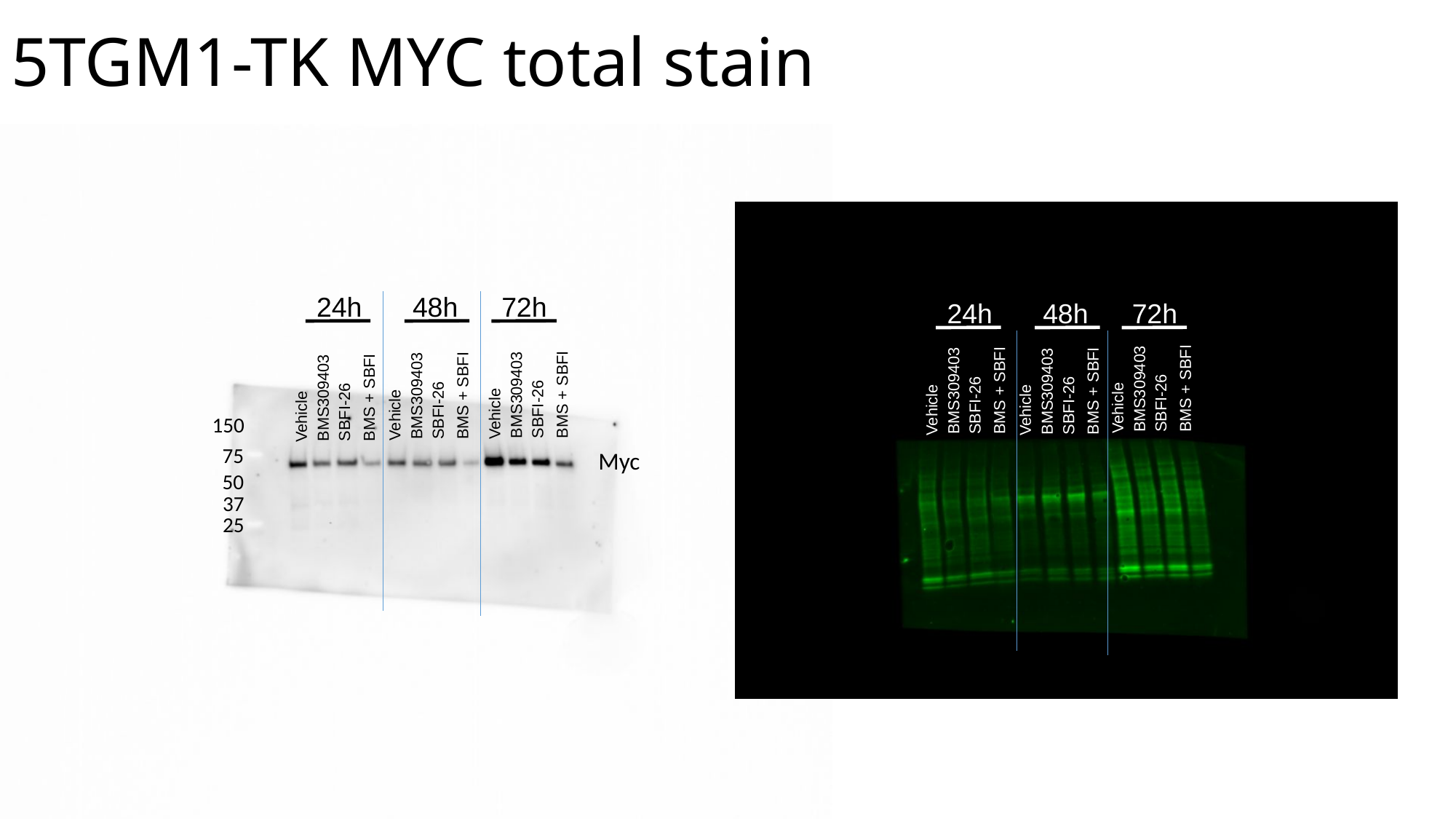

# 5TGM1-TK MYC total stain
24h
48h
72h
24h
48h
72h
BMS309403
BMS + SBFI
BMS309403
BMS309403
BMS + SBFI
BMS309403
BMS + SBFI
BMS + SBFI
BMS309403
BMS + SBFI
BMS309403
BMS + SBFI
SBFI-26
Vehicle
SBFI-26
SBFI-26
Vehicle
Vehicle
SBFI-26
Vehicle
SBFI-26
Vehicle
SBFI-26
Vehicle
150
75
Myc
50
37
25
