# Supplementary figures and images for: Targeting the fatty acid binding proteins disrupts multiple myeloma cell cycle progression and MYC signaling
Source: eLife. 2023 Mar 7;12:e81184. doi: 10.7554/eLife.81184 (PMC9995119; doi:10.7554/eLife.81184)

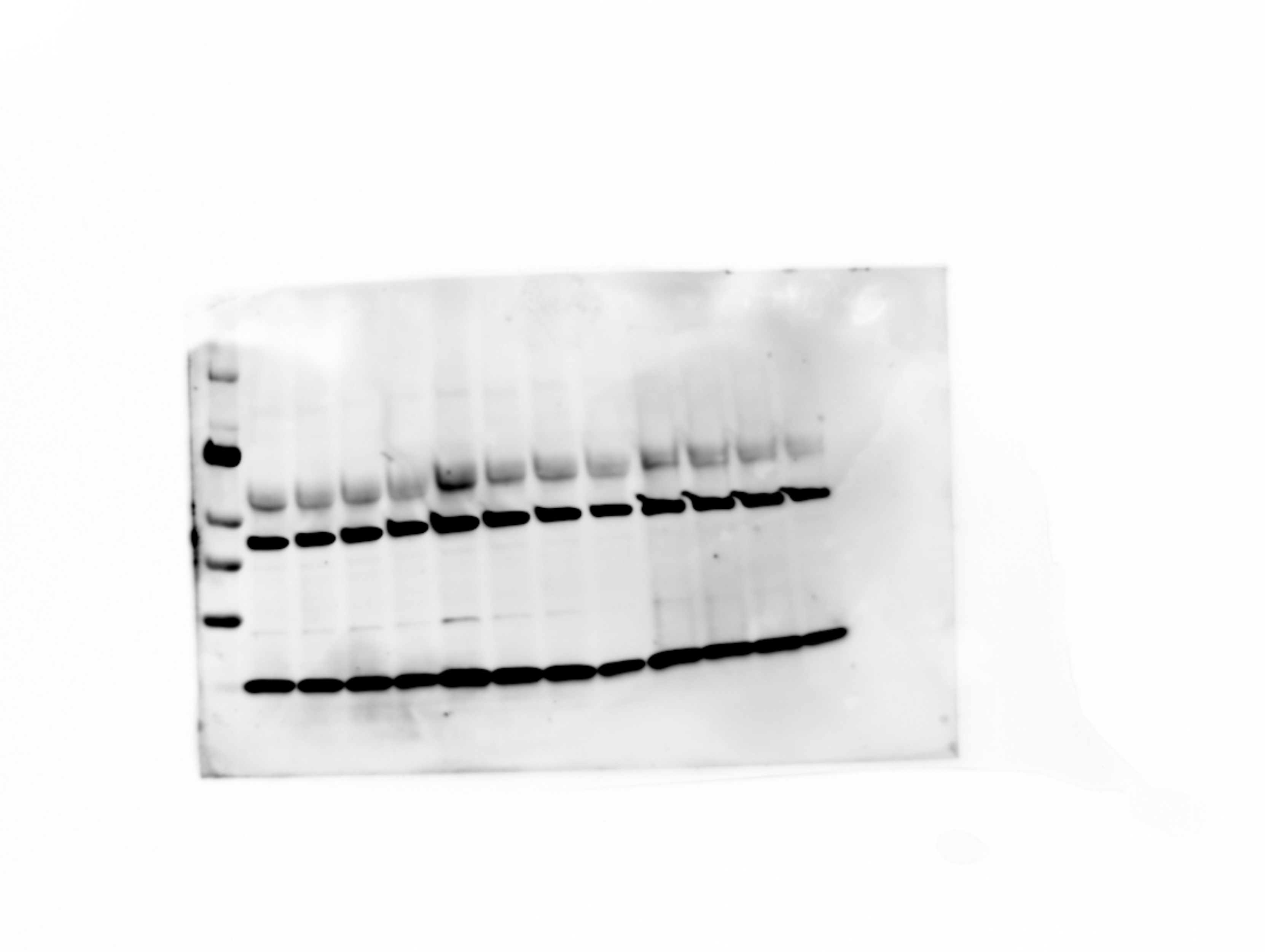

Supplement: Source data 1. [file elife-81184-data1.zip › WB/MM1S FABP5/S1F10-0116-023726_pub.tif]

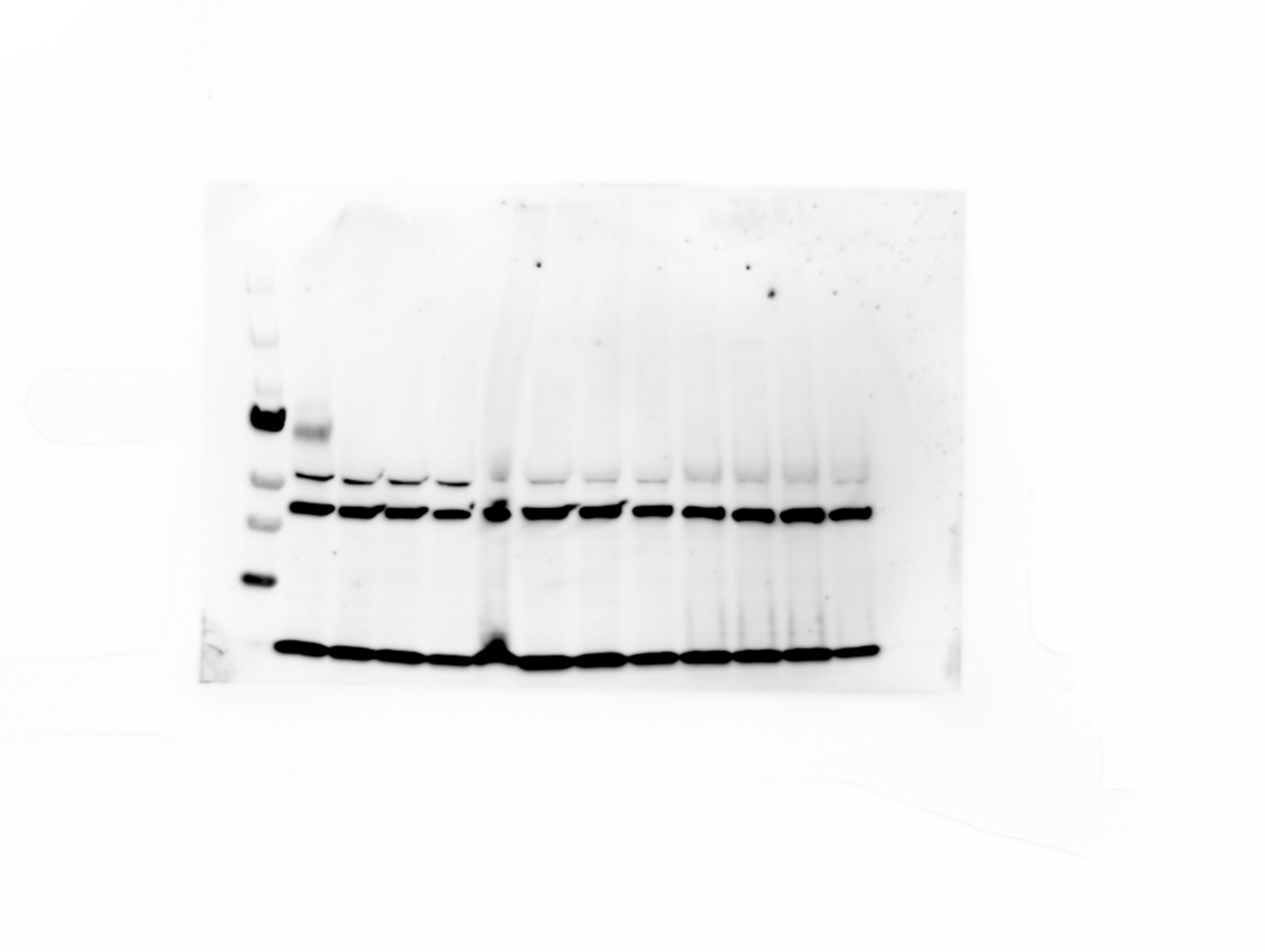

Supplement: Source data 1. [file elife-81184-data1.zip › WB/MM1S FABP5/S1F10-0117-070919_pub.tif]

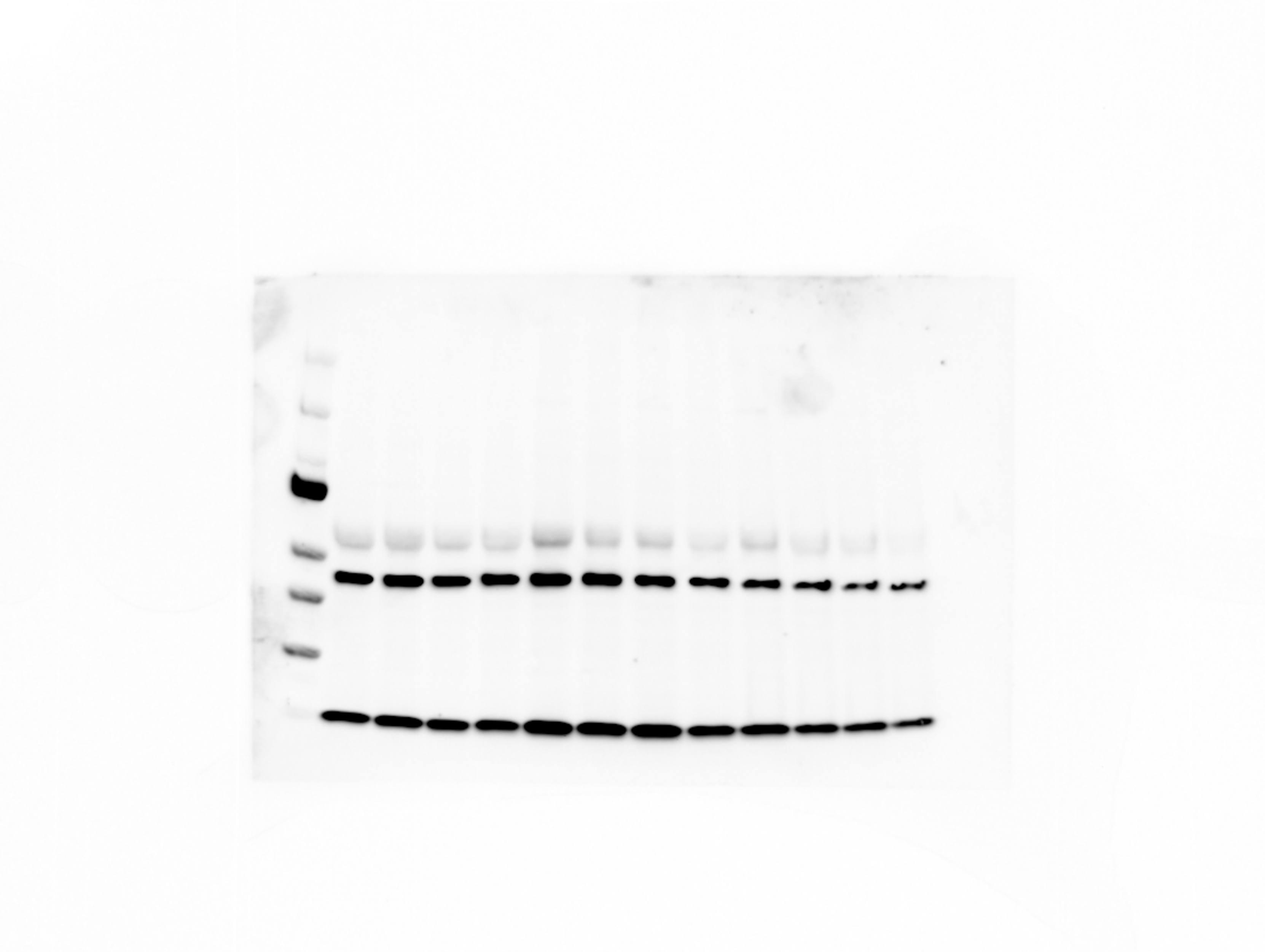

Supplement: Source data 1. [file elife-81184-data1.zip › WB/MM1S FABP5/S2F10-0116-030957_pub.tif]

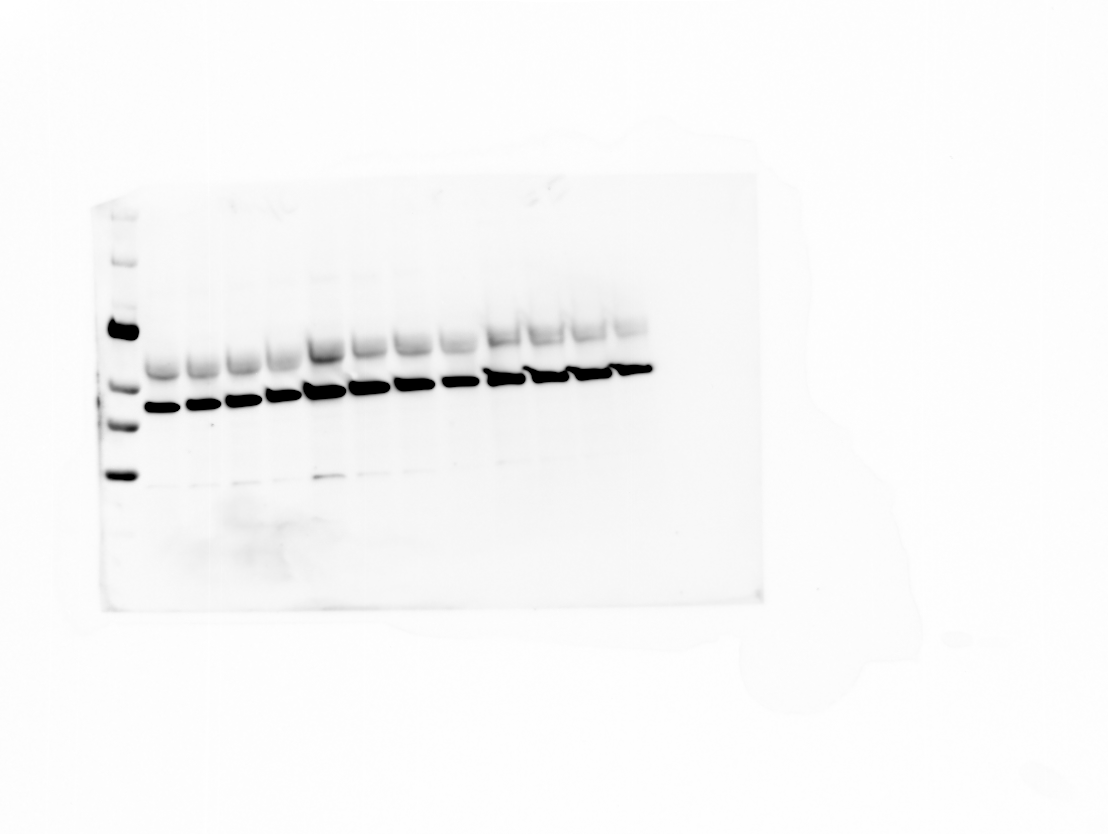

Supplement: Source data 1. [file elife-81184-data1.zip › WB/MM1S myc/S1F10-0115-021547.tif]

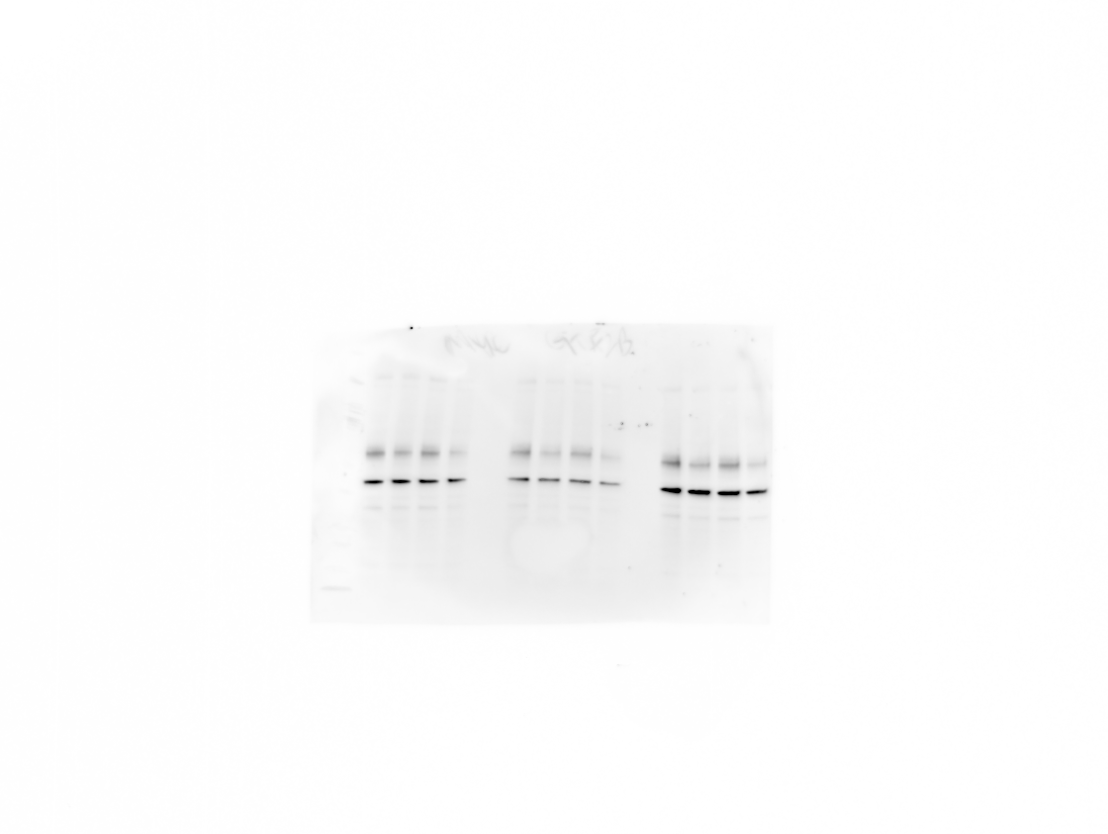

Supplement: Source data 1. [file elife-81184-data1.zip › WB/MM1S myc/S1F3-1103-231347.tif]

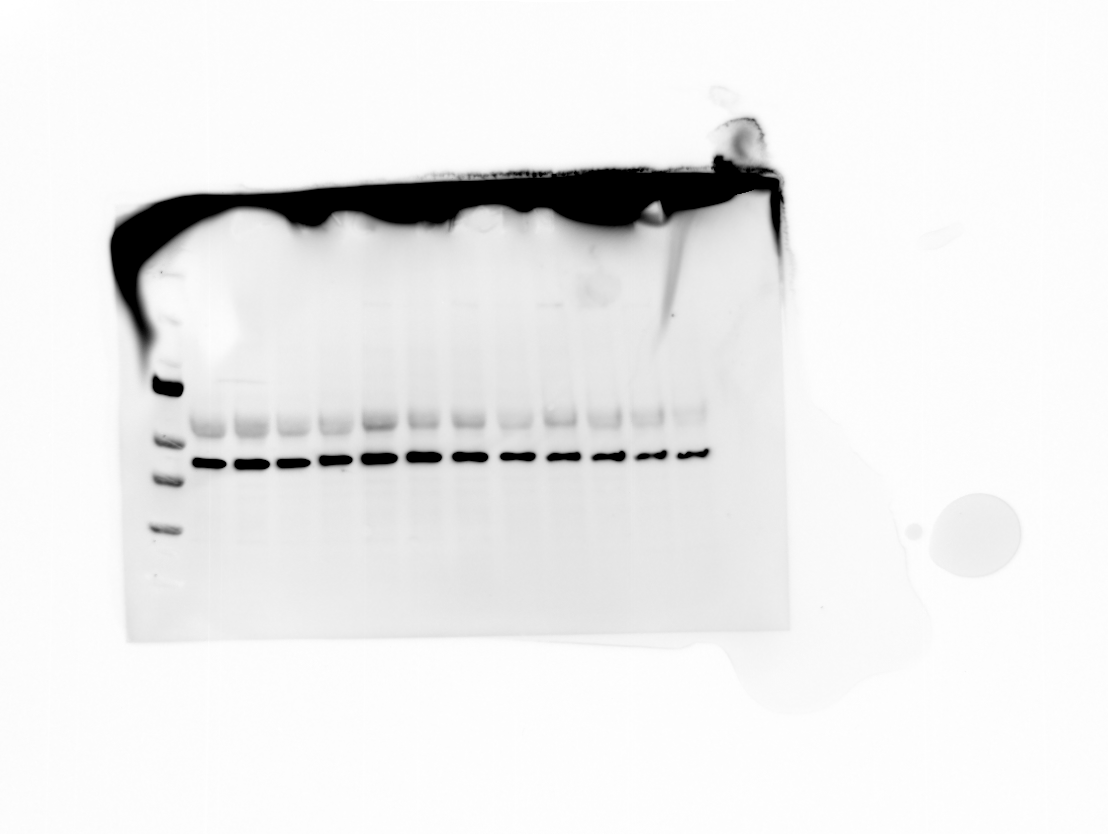

Supplement: Source data 1. [file elife-81184-data1.zip › WB/MM1S myc/S2F10-0115-024524.tif]

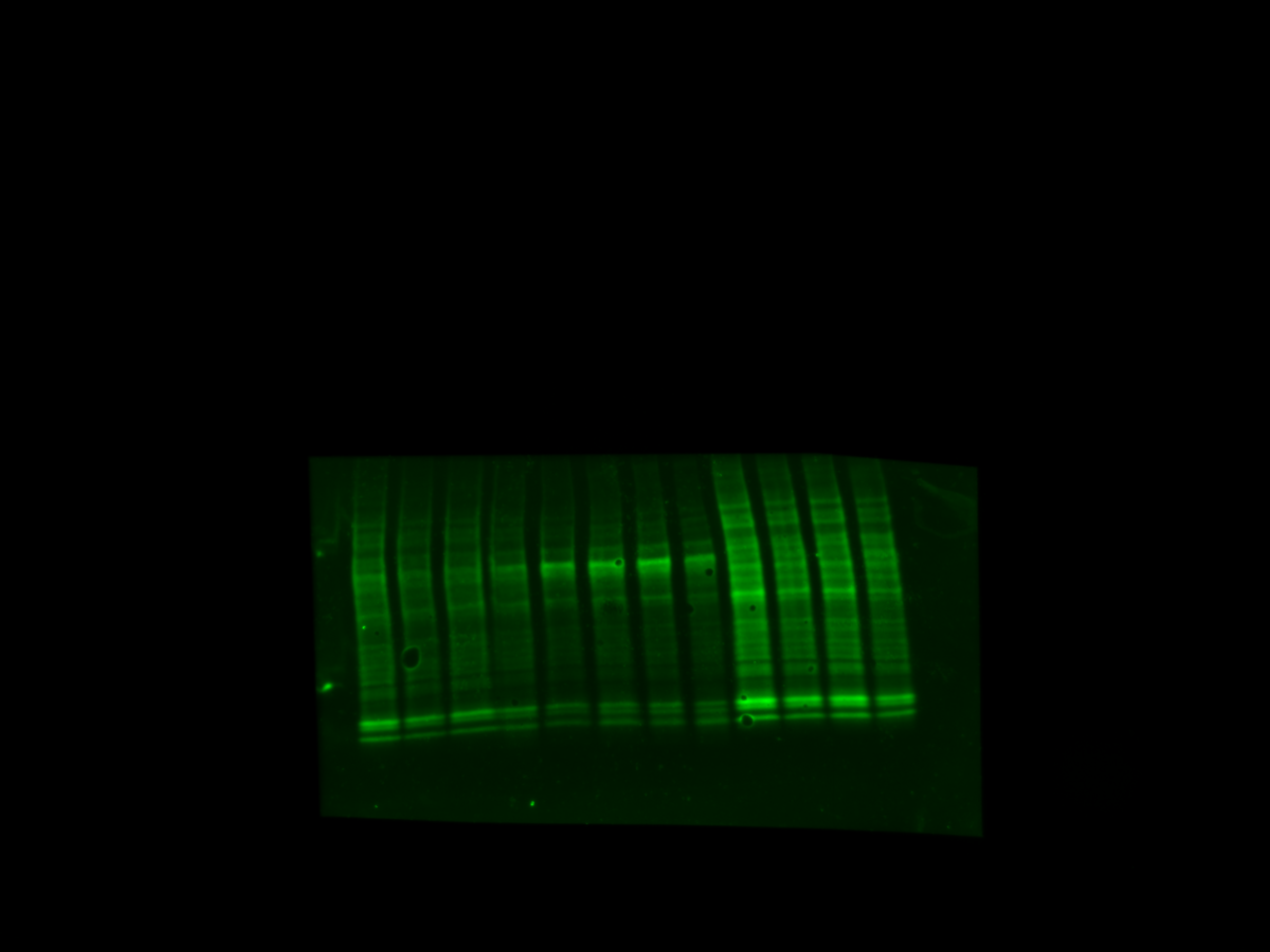

Supplement: Source data 1. [file elife-81184-data1.zip › WB/TK MYC/2022-1123-190818_pub.tif]

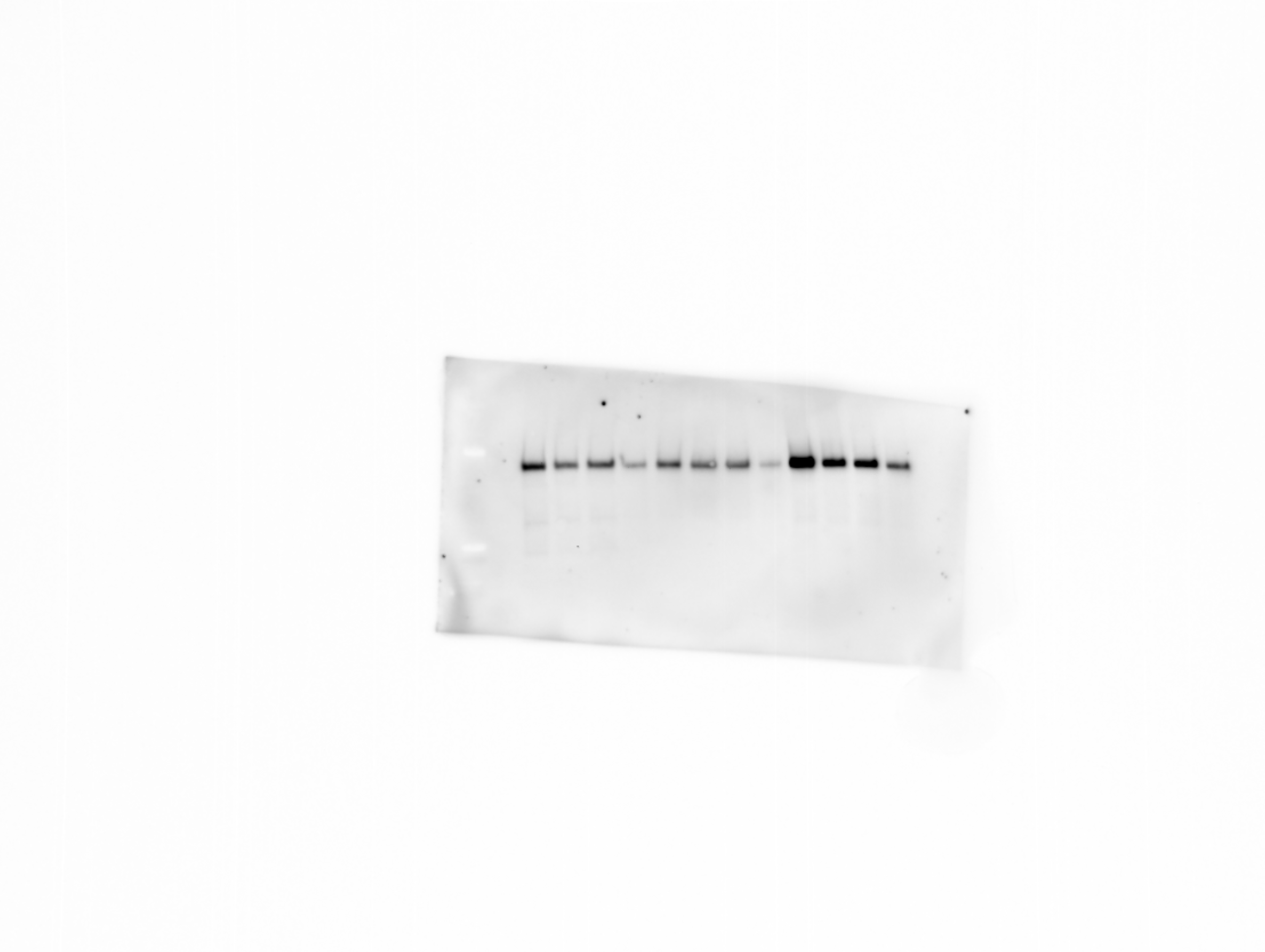

Supplement: Source data 1. [file elife-81184-data1.zip › WB/TK MYC/S2F10-1124-220253_pub.tif]
